# TB vaccine development: where are we and why is it so difficult?

**DOI:** 10.1136/thoraxjnl-2014-205202

**Published:** 2014-11-28

**Authors:** Morven E M Wilkie, Helen McShane

**Affiliations:** The Jenner Institute, University of Oxford, Old Road Campus Research Building, Oxford, UK

**Keywords:** Tuberculosis

## Abstract

The development of an effective TB vaccine remains paramount to achieving the goal of global eradication of TB by 2050. The only licensed vaccine, BCG, has variable efficacy and is poorly effective in high burden countries. The development of promising candidate vaccines to either ‘boost’ a BCG primed immune system or replace BCG altogether is a key area for innovative research. Here, we discuss some of the issues encountered in the development of potential candidate vaccines and the future challenges.

## Introduction

TB remains one of the leading global health problems of the 21st century with 8.6 million new cases and 1.3 million deaths in 2012.^1^ Approximately a third of the world's population is latently infected with the causative organism, *Mycobacterium tuberculosis* (*M. tuberculosis*), and is at risk of reactivation. The increasing burden of drug resistant TB, together with the increased susceptibility of the HIV-infected population, further confounds our ability to control this disease. The Global Plan to Stop TB: 2006–2015, established by the Stop TB Partnership, set the target for elimination of TB by 2050 with a 50% reduction in prevalence by 2015. WHO TB report 2013 highlighted that while progress is being made, it is too slow and globally we are off target with only a 2% reduction in prevalence per year.^1^

An effective vaccine is fundamental to achieving global TB control. BCG, the only licensed TB vaccine, provides protection against disseminated TB disease in children, but confers inconsistent efficacy against pulmonary disease, particularly in adolescents and young adults. BCG is contraindicated in HIV-infected adults, and with the recent change in WHO guidelines for HIV exposed infants, there is an urgent need for a safe and more effective vaccine. Three questions that we need to ask are: (1) Can we break the transmission cycle to reduce disease burden? (2) Can we develop a safe and effective vaccine for everyone including HIV-infected individuals? (3) Is there a potential role for a vaccine in the treatment of drug resistant TB as an adjunct to chemotherapy?

## Vaccine development and challenges

**Figure 1:**
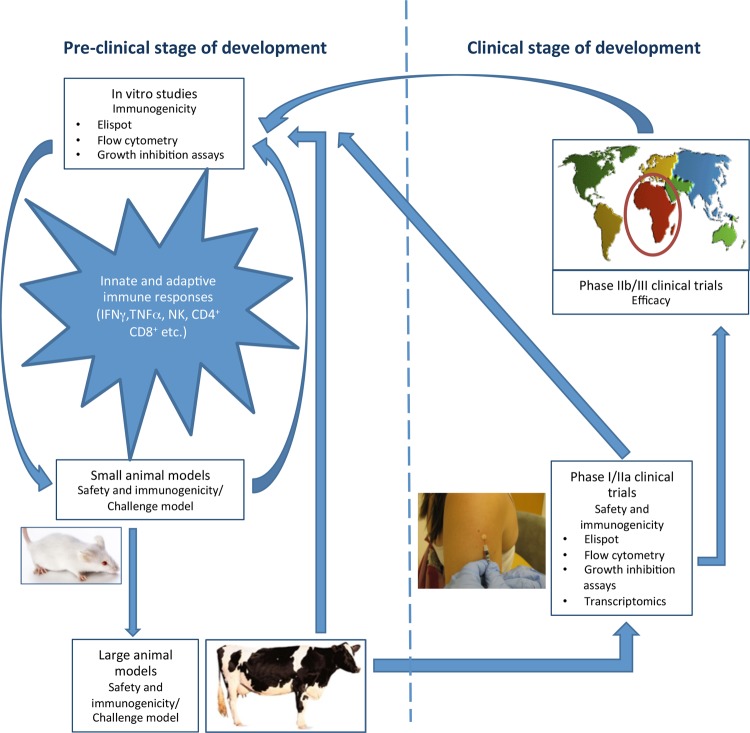
Pathway for TB vaccine development: successful candidate TB vaccines from preclinical stages of development progress to phase I/IIa safety and immunogenicity trials. Once safety and immunogenicity are demonstrated, they then move to phase IIb/III large scale efficacy trials in target populations. Efficacy, safety and immunological data from these trials then feed back into the preclinical *in vitro* and animal challenge models to aid in development of more efficacious vaccines.

TB vaccine development begins with an understanding of the type of immune response required for protective immunity. For many vaccine preventable infectious diseases, protection is conveyed through attaining sterilising immunity either from natural exposure to the pathogen or by vaccine induced neutralising antibody production. We know that exposure to *M. tuberculosis* does not induce sufficient protective immunity; however, not enough is known about the complex nature of the innate and adaptive human cellular immune responses central to natural clearance of *M. tuberculosis* infection, knowledge of which would significantly facilitate TB vaccine development. As such, candidate vaccines are assessed in preclinical animal models for safety, immunogenicity and efficacy against virulent *M. tuberculosis* challenge. Successful candidates then progress to phase I, first-time-in-human clinical trials, where safety and immunogenicity are evaluated. Following further trials in target populations to optimise dose and regimen, promising candidates progress to phase IIb/III efficacy trials (figure 1).

Identifying which vaccines should progress onto large, expensive and time consuming field efficacy trials is extremely challenging. While we know a robust Th1 cellular immune response is essential for protection, we do not have a validated correlate of protection with which to select vaccine candidates. In addition, the role of antibodies in TB vaccine induced protection, key to the development of licensed vaccines for use against other infectious diseases, has not yet been determined. Therefore, the complexity of the immune mechanisms involved in protective immunity to *M. tuberculosis* infection and the absence of an easily identifiable correlate of protection such as those quantifiable in antibody-mediated diseases demonstrate the unavoidable empiricism with which candidate vaccines are developed. Subsequently, we do not know which of the animal models best reflect human disease, and these models cannot be validated until we have an effective vaccine in humans.

It is clear that with these uncertainties, more research is needed. A blueprint for the TB vaccine development pipeline, encompassing the next decade, has been outlined with the simple vision: ‘to introduce the safest and most effective TB vaccines that reduce tuberculosis worldwide through partnerships, innovative strategies and creative mechanisms’.^2^ Five key areas have been identified: (1) innovative and creative research and development, (2) identification of correlates of immunity and biomarkers for TB vaccines, (3) the harmonisation and cooperation of invested groups/organisations, (4) rational selection of TB vaccine candidates and (5) the critical need for advocacy, community acceptance and available funding.^2^

## Lessons learned from current candidates

Currently, there are 12 candidate vaccines in development.^2^ These candidates are designed either to replace BCG or to boost it. One of the most clinically advanced candidates is MVA85A, a recombinant strain of modified vaccinia virus Ankara expressing the conserved *M. tuberculosis* antigen 85A. MVA85A was designed to deliver a heterologous ‘boost’ to a BCG primed immune system and to boost high levels of antigen specific T cells. Preclinical studies in animal models showed that boosting BCG with MVA85A could enhance protection against an *M. tuberculosis* challenge. Human phase I/IIa studies demonstrated MVA85A to be safe and immunogenic and to induce antigen specific Th1 and Th17 cells, both considered important in protection against TB. In 2009, MVA85A was the first subunit TB vaccine to enter into a phase IIb efficacy trial in BCG-vaccinated South African infants. For the primary study outcome of safety, MVA85A was well tolerated with an acceptable safety profile.^3^ However, MVA85A did not significantly improve on BCG alone in terms of protection, either against TB disease or *M. tuberculosis* infection. Immunological analysis revealed only modest antigen specific T cell responses, which were 10-fold lower than those seen in UK adults. Despite this, much can be learned from this trial. The time and money involved in these field efficacy trials are substantial, and it is essential that we use the information gained from this study to iteratively refine our vaccine selection process. Reviewing the preclinical efficacy data for MVA85A demonstrates that the improvement in efficacy in four preclinical models did not predict efficacy in humans. This may be in part because human efficacy trials are powered to show a much larger effect than we see in animals.^4^ A review of the design of preclinical animal models in an attempt to make them more predictive is required.

The immunological data also merit review. What impact does the immature immune system of infants have on the immunogenicity of new vaccines, and are we targeting the wrong cohort? As adolescents and adults are primarily responsible for the transmission of TB, is this age group a more important target population to evaluate efficacy? Ongoing work on immune correlate samples taken from all infants in this trial may reveal biomarkers to guide vaccine development.

Finally, human challenge models have been invaluable in malaria vaccine development; however, development of a human mycobacterial challenge model is complex. Attempts are being made to use BCG in a human challenge model, which might then be used together with immunological and preclinical efficacy data to guide vaccine selection.^5^

## Summary

In the last decade, significant progress has been made in TB vaccine research. Many candidate vaccines have been evaluated in clinical trials in high burden countries. However, the challenges for the next decade are clear. The refinement of the preclinical animal models, together with the identification of immune correlates of protection, would greatly facilitate vaccine development. Given the uncertainties in this field, a collaborative approach between vaccine researchers and developers is essential if progress is to be expedited. It is essential that we maintain the momentum gained over the last decade if we are to have a new licensed vaccine in the next decade.
